# A Web-Based Registry Framework Linking Transfusion Practice to Long-Term Patient Outcomes: Design and Single-Center Feasibility Study

**DOI:** 10.1007/s10916-026-02474-2

**Published:** 2026-10-21

**Authors:** Elina Nürenberg-Goloub, Benjamin Friedrichson, Michael Nordine, Holger Storf, Kai Zacharowski, Jan Andreas Kloka

**Affiliations:** 1 Department of Anaesthesiology, Intensive Care Medicine and Pain Therapy, Goethe University, University Hospital Frankfurt, Frankfurt, Hessen, Germany; 2 University Medicine, Institute of Medical Informatics, Goethe University, Frankfurt, Germany

**Keywords:** Health Information System, Interoperability, Benchmarking, Patient-Reported Outcome Measures (PROMs), Transfusion Registry

## Abstract

Blood transfusions are among the most common medical interventions performed worldwide. Several comprehensive databases link transfusions to routinely collected health data but do not include the indication and the decision-making basis for a particular administration, nor patient-reported long-term outcomes. We developed a web-based registry for the standardized assessment of transfusion practice and patient outcomes, designed for an observation period of up to 30 years. The framework combines clinical data captured at the point of care with patient-reported outcome measures collected through automated digital follow-up, ensures privacy by design through physical separation of identifying and medical data, and codes diagnoses and procedures in SNOMED CT. We evaluated feasibility in a single-center pilot at Goethe University Hospital Frankfurt (11 September 2024–15 January 2026), with patient-reported follow-up collected through 13 August 2026. A total of 408 patients were enrolled, yielding 1,298 administrations across nine treatment categories in a predominantly cardiac surgical cohort. The pilot demonstrated that the registry integrates into routine clinical workflows, captures the parameters required for guideline-adherence monitoring, and supports sex-disaggregated, descriptive analyses of transfusion practice. Follow-up was completed by 12.5% of patients at 3 months and 15.2% of patients at 1 year; paired baseline–follow-up assessments of physical capacity and frailty demonstrate the data structure for capturing functional recovery. Risk-adjusted benchmarking, network-wide analyses, and donor outcomes are designed capabilities of the framework that were not evaluated in this pilot. The platform is available in 16 languages and participation is open to further institutions.

**Supplementary Information:**

The online version contains supplementary material available at https://doi.org/10.1007/s10916-026-02474-2.

## Introduction

Blood transfusions are among the most common medical interventions performed globally. Nevertheless, it remains difficult to link individual transfusion decisions to their clinical context and long-term patient outcomes, which limits the monitoring of clinical practice and its impact on patients’ lives. Such monitoring is needed: despite the known risks to patients and the heavy burden on healthcare systems, there are still unwarranted variations in transfusion practice between individual doctors, institutions and countries [1–5]. In a recent international cohort of 3,643 patients across 233 intensive care units in 30 countries, the proportion of patients who received packed red blood cells (RBC) ranged from 0 to 100% across centers and from 19 to 45% across continents [6]. In elective major surgery, RBC transfusion rates varied from 2 to 72% across 61 centers and 23–99% of them were inappropriate according to clinical guidelines [7]. Variation and uncertainties also exist for blood components like fresh frozen plasma (FFP) and platelets [8, 9] and blood-derived products like human serum albumin [10], or fibrinogen [11]. Notably, the number of acute transfusion-associated adverse events is substantially underreported [12]. Active haemovigilance shows that transfusion-associated circulatory overload (TACO) alone affects 2.2% of all recipients (pooled estimate; up to 27% of recipients in individual studies) [13] with an in-hospital mortality of 21% (compared to 11% in matched transfused patients) [14]. In contrast, only 215 TACO cases and 11 deaths (total: 3,170 serious adverse reactions) were reported to the European Commission in 2023, against 2.9 million recipients of 16 million units of blood components [15]. In the United Kingdom, SHOT reported 188 TACO cases and 31 deaths (total: 676 adverse reactions) against 2.2 million components issued in 2024 and acknowledged underreporting and gaps in diagnosis and treatment of such complications [16]. While justified transfusions are undoubtedly effective in saving lives, unnecessary transfusions have the potential to pose significant risks to patients and result in increased expenditures throughout the entire blood product supply chain. Moreover, they can also lead to significant medical complications [17, 18]. Additionally, in periods of global blood shortage, the unwarranted administration and wastage of blood components undermine the ethical contract with voluntary donors [19, 20]. Supporting hospitals in the ongoing assessment and improvement of their own transfusion practices directly at the point of care, rather than relying on retrospective national reports, is therefore a clinical and ethical imperative.

Beyond the acute setting, alloimmunization against non-AB0 blood group antigens occurs in 2–5% of all RBC recipients [21]. For multiply transfused patients, each exposure increases cumulative risk of adverse reactions. For women of childbearing age, alloantibodies can cause hemolytic disease of the fetus and newborn [22]. Ethnic minorities are disproportionately affected because blood group antigens are not equally distributed across populations, and underrepresentation in donor pools limits the availability of antigen-matched products [23].

Large infrastructures now connect transfusion exposure, donor and component data, health records, and longitudinal outcomes at scale [24–26]. The Dutch PROTON study, linking 290,000 transfusion recipients to national mortality records, found life expectancy reduced by 4–15 years depending on the blood component received [24]. The Scandinavian SCANDAT database, tracking over 1.1 million recipients, documented elevated mortality ratios persisting 15–17 years after transfusion [25]. Whether these associations are causal or reflect confounding is unresolved due to the retrospective designs and the lack of detailed clinical context, exact indications, and dosing.

To address these gaps, we designed the Registry Study for Research of Blood Donor and Recipient Long-Term Outcomes (DRKS00034405; hereafter “the registry”) as a digital framework for standardized assessment of transfusion practice and patient outcomes over up to 30 years. The registry combines SNOMED CT-coded clinical data documented by medical professionals at the point of care with patient-reported outcome measures (PROMs) collected through automated digital follow-up. Privacy by design is ensured through separation of identifying and medical data, and participating institutions share the scientific governance of the registry. Here, we present the design rationale, the digital data collection framework, and the first results of our single-center feasibility study at the Goethe University Hospital in Frankfurt, Germany.

## Methods

### Setting

The feasibility study was conducted at Goethe University Hospital Frankfurt, a tertiary academic center. Enrollment was concentrated in the cardiac surgery program; the pilot cohort is therefore predominantly cardiac surgical, which should be considered when interpreting the results.

### Research Items Development and Data Collection Design

A digital registry based on the open-source registry software solution OSSE was implemented for data collection [27]. The data elements and follow-up schedule were defined by two clinicians (JK, MN) and independently refined by two other clinicians (BF, KZ), prioritizing validated instruments feasible for repeated self-administration over up to 30 years. Patient-reported follow-up questionnaires were designed for completion in approximately five minutes by individuals with low medical literacy, using layered filter systems and optional responses throughout to minimize respondent burden and dropout over the planned 30-year observation period.

The registry captures data across three electronic case report forms (eCRFs): (i) basic demographics and (ii) transfusion/blood product entry are embedded in a single graphical user interface (GUI) and completed online by medical staff at the treatment center. Diagnoses and procedures are recorded as SNOMED CT concepts by medical staff, who can browse them using English keywords. The (iii) PROMs are provided upon email request with a personalized token in an online GUI accessible without a password from any device.

### Outcome Measures

In the clinical episodes, medical staff records (i) variable biometric data (weight, height, sex), (ii) primary and secondary diagnoses, (iii) physical capacity, graded in five activity-anchored metabolic equivalents of task (MET) categories based on the recognized Duke Activity Status Index [28, 29], (iv) frailty using the Clinical Frailty Scale (CFS) as a validated instrument for long-term follow-up [30, 31], (v) blood group antigens AB0 and RhD, (vi) documentation of blood product and hematology medication (date, relevant laboratory values before and after administration, specific indications, and dosages), (vii) details on the clinical intervention, including the patient-specific hemorrhage risk based on the anesthesiologic assessment (low: <500 ml; high: ≥500 ml) and the day of hospital discharge. In-hospital mortality was not captured in the pilot eCRF version; it has since been added to the basic data form of the current eCRF iteration.

Long-term outcomes captured through PROMs are (i) newly diagnosed diseases, as indicators of long-term morbidity [24, 25]; (ii) anemia diagnosis and treatment, as indicators of persistent or recurrent anemia; (iii) receipt of blood products or hematologic medication in the past year and related adverse events, capturing re-exposure and late complications [13, 14, 21]; (iv) pregnancies and births, relevant for alloimmunization effects [22]; and (v) functional status by MET and CFS (with clear explanations), enabling comparison with the clinician-documented baseline. No question is mandatory with the possible option “I do not know / do not want to answer” to avoid discomfort.

### Legal and Ethical Framework

Patients are enrolled in the study based on informed consent. The General Data Protection Regulation (GDPR) and the Declaration of Helsinki apply. Participants may withdraw consent at any time. The OSSE framework ensures privacy by default through physical separation of identifying data (IDAT) and medical data (MDAT) [27]. The study was approved by the Ethics Committee of the Goethe University Hospital Frankfurt (approval number 2024 − 1720_2, Chair: Prof. Dr. Harder) and registered in the German Clinical Trials Register (https://drks.de/) on 10 July 2024 and the WHO International Clinical Trials Registry Platform (https://trialsearch.who.int/) under the number DRKS00034405.

### Inclusion and Exclusion Criteria

**Fig. 1:**
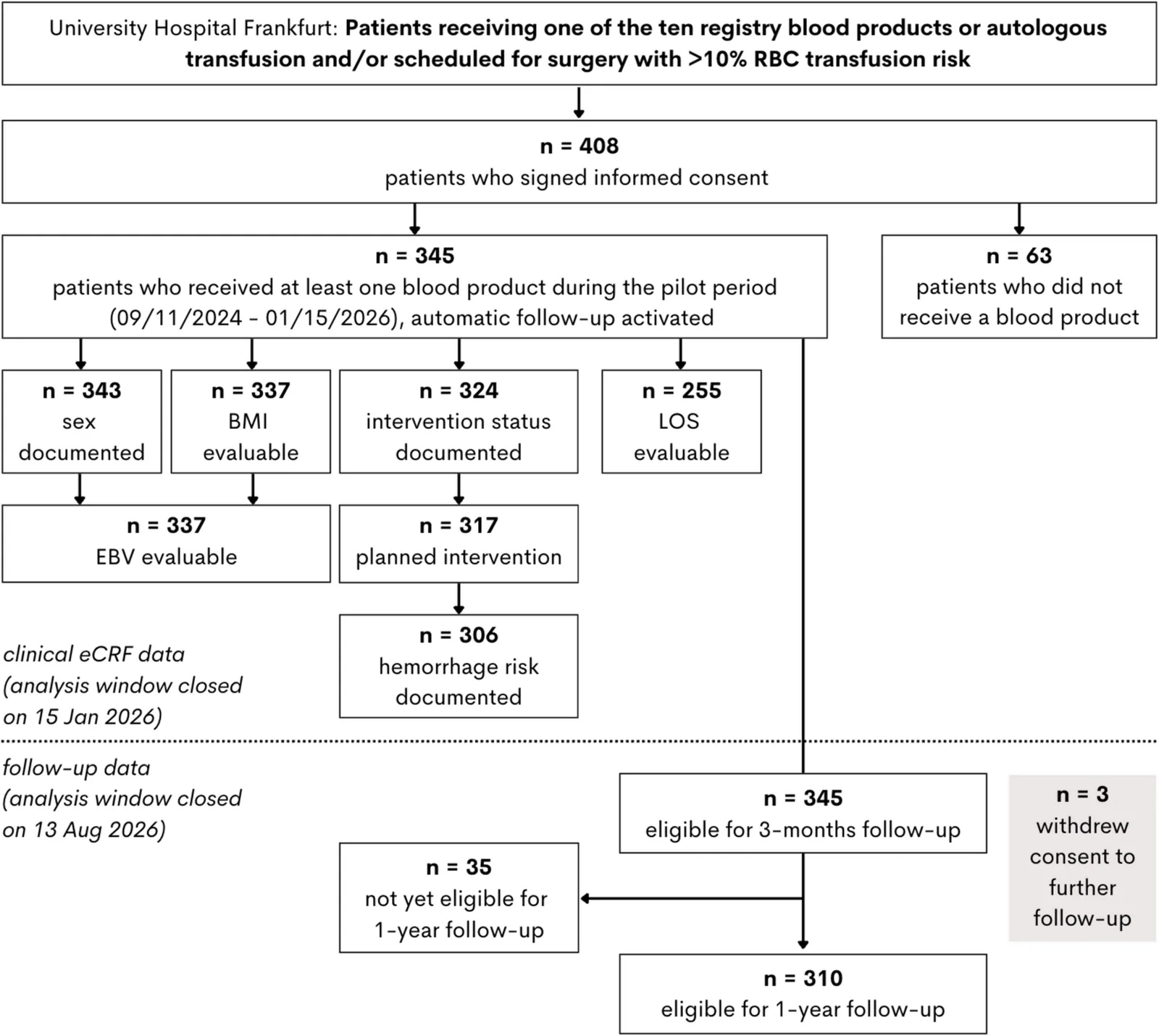
Patient inclusion scheme for the feasibility study analyses. Patients who did not provide informed consent were excluded while those who consented but did not receive a blood product until the database export remain registered. PROMs were counted for the pilot population until 13 August 2026. Three patients withdrew consent to further follow-up; their clinical data remain in the analysis, and they were retained in the follow-up denominators as non-responders. EBV, estimated blood volume; LOS, post-interventional length of hospital stay

All patients who receive one of the ten listed blood products captured by the current version of the registry (RBC, FFP, platelet concentrate, fibrinogen, albumin, prothrombin complex concentrate/PCC, antithrombin III, intravenous and oral iron, erythropoietin) and/or autologous transfusion and/or are scheduled for a surgical intervention with high RBC transfusion risk (> 10% based on anesthesiologic assessment of the patient and planned procedure [32]) and provide informed consent personally or via a legal representative at any timepoint during their hospital stay are included in the study (Fig. 1). The only exclusion criteria for the study are the inability or refusal to provide informed consent.

### Data Analysis and Visualization

All statistical analyses were performed using Python 3.12 with pandas 2.3.0, numpy 2.0.0, and matplotlib 3.10.0. The modular analysis pipeline was developed using synthetic data in collaboration with the AI assistant Claude Opus 4.5 (Anthropic PBC, San Francisco, USA). Synthetic data were based on *n* = 8 pseudo-patients entered manually into a parallel test registry eCRF, then expanded by the AI assistant to *n* = 100 pseudo-patients using the data dictionary with clinically plausible ranges defined by physicians (JK, MN). The synthetic dataset deliberately included common data quality issues to ensure robust error handling and flagging for manual review (missing values, out-of-range entries, unit and dosage inconsistencies, follow-ups outside eligibility windows and follow-up duplicates). Scripts were generated through iterative development: (i) a script for statistical analysis and/or visualization was developed with the AI assistant on the basis of synthetic data, (ii) if an error occurred during the execution with real data, the source was manually inspected and similar data quality issues were introduced into the synthetic dataset (iii) the script was further developed with the AI assistant to ensure that it will robustly handle the issues, (iv) steps i-iii were repeated until all analysis modules could be completed without errors for the real data. Real patient data were not entered into, processed by, or exposed to any AI system. The analysis pipeline was first validated comparing its results for miniature datasets with manual calculations (Excel, Microsoft, Redmond, USA). Second, we compared its results for synthetic datasets (*n* = 100, *n* = 150) that included deliberate errors with the known answers. Final analyses were performed by the investigators using the validated scripts in a closed virtual environment.

Figures were generated using a colorblind-friendly palette [33]. Violin plots display Gaussian kernel density estimates with bandwidth selected by Scott’s rule and embedded box plots. Outliers in box plots were identified using Tukey’s method (values beyond 1.5 times the IQR from Q1 or Q3) and displayed as individual points.

### Follow-up Eligibility

Patient-reported follow-up was counted through 13 August 2026 but restricted to the pilot cohort (patients that had ≥ 1 blood product record until 15 January 2026). Follow-up is anchored to the date of the first administration, with response windows of 68–113 days (3-month) and 273–455 days (1-year). Patients were eligible for follow-up if their window had opened by 13 August 2026 (Fig. 1).

### Statistics

Descriptive statistics were calculated at two levels: administration-level (each blood product administration as unit of analysis) and patient-level (using available values for baseline characteristics). All analyses are descriptive. Continuous variables are presented as mean ± standard deviation (SD) and median with interquartile range [IQR]. Categorical variables are presented as frequencies and percentages. Sex was recorded in the eCRF under the German field label “Geschlecht” with the options male, female, and diverse. German does not lexically distinguish biological sex from gender. All sex-stratified analyses refer to sex as documented by the medical staff. Diverse entries, had they occurred, would have been reported as a separate category. Age was estimated from year of birth to the date of the clinical episode since the day of birth is recorded as IDAT and is not available for research; this may introduce an error of up to one year. Body mass index (BMI) was calculated as weight (kg) / height squared (m²); values outside the range of 10–60 kg/m² were excluded. Post-interventional length of hospital stay (LOS) was calculated from surgical intervention to discharge; negative values and hospitalization for more than 365 days were excluded from the analysis. Diagnoses and procedures for the most common concepts in the total cohort and the RBC recipients were manually retrieved from the SNOMED International browser (https://browser.ihtsdotools.org/*).* For RBC and FFP, dosages were normalized to estimated blood volume (EBV) calculated using Nadler’s formula [34]. For other products, body weight (kg)-adjusted dosages were calculated. For dosage analysis, implausible values (e.g. unit entry errors) were identified by clinical review; details are reported in the respective table legends. Laboratory value changes were calculated as the difference between pre- and post-administration values for paired measurements. Laboratory values were screened for clinical plausibility against physician-approved (JK, MN) reference ranges. Entries documented in non-standard units were converted if possible and entries outside the clinically plausible ranges were excluded (see table legends). AB0 blood type compatibility assessment (RBC, FFP, platelet concentrate) required documentation of patient and product blood types. Data completeness was assessed as the proportion of non-missing values for each variable. Follow-up response rates are unique responding patients divided by eligible patients. Identical duplicate records were resolved by retaining one; conflicting duplicates were excluded. Paired comparisons always refer to the respective follow-up versus baseline.

## Results

### Registry Design

**Fig. 2:**
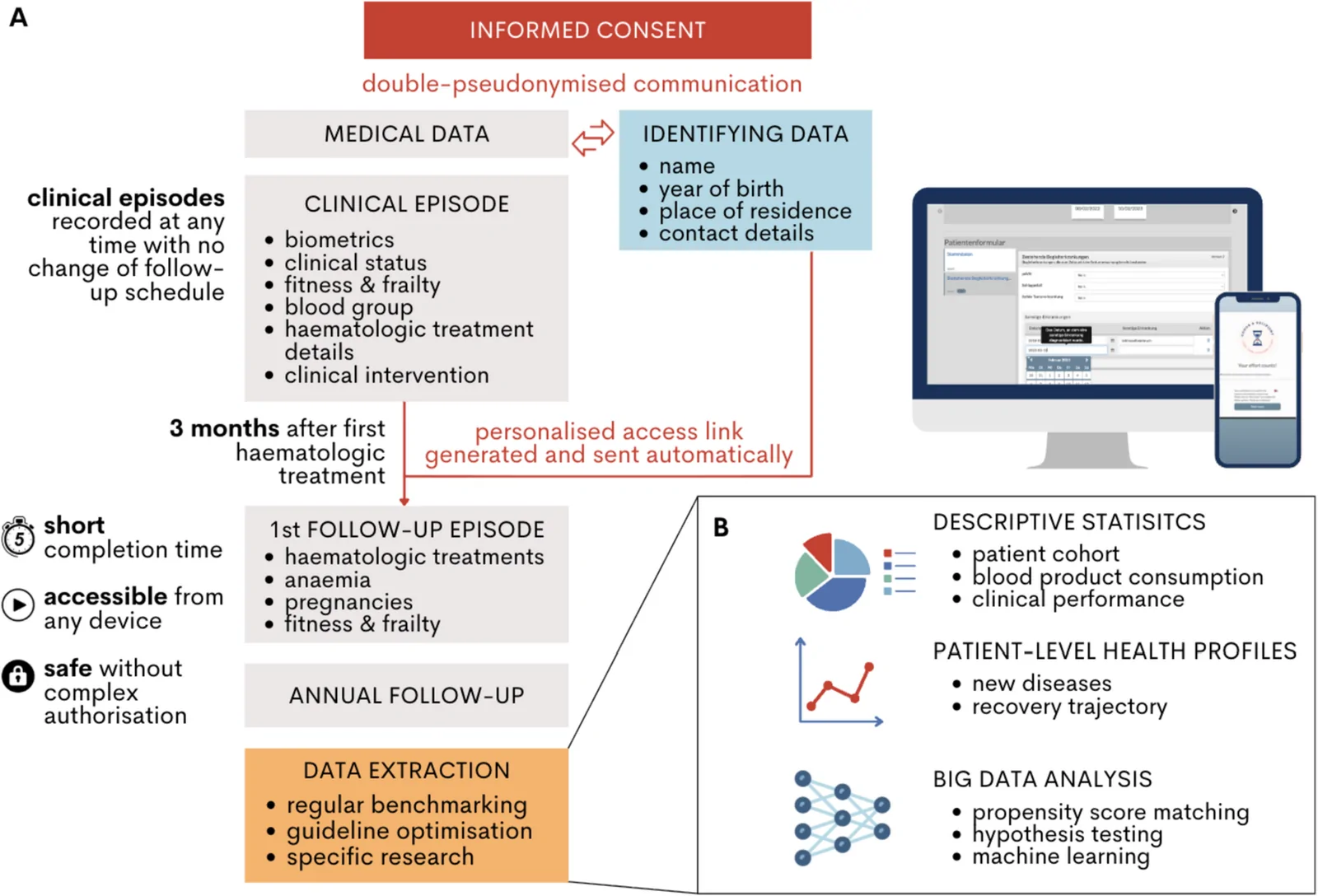
Registry data collection workflow and analytical outputs. (**A**) Data collection architecture. Following informed consent, medical and identifying data are stored separately using double-pseudonymized communication. Medical staff records clinical episodes at any time without affecting the follow-up schedule. Three months after the first hematologic treatment, patients receive a personalized access link to complete the first follow-up questionnaire, followed by annual follow-up. The digital platform ensures short completion times, device-independent accessibility, and secure data handling without complex authorization procedures. (**B**) Analytical outputs at three levels: institutional benchmarking, patient-level health profiles, and network-wide big data analyses

The design and implementation of the registry were guided by the European Medicines Agency Guideline for Registry-Based Studies and the US Agency for Healthcare Research and Quality Guide for Patient Outcomes Evaluation [35, 36]. The registry is a web-based application accessible to physicians from any device with internet connection for patient enrolment. For follow-up, a single-use personalized link is automatically sent to the patient’s contact email (Fig. 2). The pilot version was implemented in German and English, and is now expanded to 16 European and Asian languages. All diagnostic and procedural data are coded using SNOMED CT, supporting standardization within the registry and reusability in accordance with the FAIR data principles. The centralized OSSE eCRF ensures that records from all hospitals can be analyzed as a single dataset but stay protected by double pseudonymization via the Mainzelliste [37]. Structured data exchange via HL7 FHIR is planned for the underlying OSSE platform but not yet implemented (Table S1). All participating institutions serve as equal partners in the scientific and governance bodies of the project.

### Research Items and Evaluation Scheme

In accordance with the principle of data minimization, the patient is registered using a small IDAT set (name, year of birth, city of residence, contact email). The medical data collection framework covers multiple assessment domains (see Methods). Medical staff can capture any number of clinical episodes at any point in the lifetime of the patient. An initial follow-up episode at three months after the first blood product or drug administration, followed by annual assessments yields longitudinal patient-level health profiles (Fig. 2).

The registry supports three levels of analysis: (i) descriptive statistics; (ii) patient-level health profiles; and (iii) network-wide big data analyses (Fig. 2). It is designed to provide participating institutions with customizable benchmarking reports comparing their transfusion practices against anonymized aggregate data from the registry network. Additionally, we aim to develop institutional dashboards with real-time updates, enabling timely quality improvement opportunities. These analytical capabilities beyond the descriptive institutional level are designed but not yet evaluated (Table S1).

### Results of Piloting Phase

During the piloting period (11 September 2024 to 15 January 2026), a total of 408 patients were enrolled in the study and recorded in the registry. Most patients (84.6%, *n* = 345) received at least one blood product administration, while 63 patients (15.4%) were registered but did not receive relevant therapy within the pilot period. No patient was recorded as diverse; sex was undocumented for two patients. Of 343 patients with documented sex, 245 (71.4%) were male and 98 (28.6%) were female (Table 1). A surgical intervention was planned for 97.8% of the patients with documented status (*n* = 317/324). The expected hemorrhage risk was high (≥ 500 ml expected blood loss) in 89.9% of the patients with a documented risk category (*n* = 275/306). Accordingly, 97.7% of the blood product administrations with documented status were connected to a planned intervention (*n* = 1,145/1,172) and 96.1% (*n* = 1,064/1,107) to a high hemorrhage risk (Figure S1). Mean LOS was 10.8 ± 10.2 days with a median of 7 days [IQR 6–11] for 255 patients with documented intervention and discharge dates. Sex-stratified analysis shows similar mean LOS for males (10.9 ± 11.5 days, median 7 [6–10], *n* = 178) and females (10.5 ± 6.4 days, median 8 [6–12], *n* = 77).

**Table 1:** Characteristics of the feasibility study cohort. BMI, body mass index; IQR, interquartile range; LOS, post-interventional length of hospital stay; SD, standard deviation

Characteristic	Overall (*n* = 345)	Male (*n* = 245)	Female (*n* = 98)
Demographics			
Age, years, mean (SD)	64.2 (11.2)	64.3 (10.8)	63.7 (12.2)
Age, median [IQR]	66 [58–72]	65 [58–72]	66 [57–73]
BMI, kg/m², mean (SD)	26.7 (4.8)	27.0 (4.1)	25.8 (6.1)
BMI, median [IQR]	25.8 [23.5–29.3]	26.2 [24.3–29.5]	24.1 [21.8–27.7]
LOS	(*n* = 255)	(*n* = 178)	(*n* = 77)
LOS, days, mean (SD)	10.8 (10.2)	10.9 (11.5)	10.5 (6.4)
LOS, median [IQR]	7 [6–11]	7 [6–10]	8 [6–12]
Treatment intensity			
Administrations, total	1,298	941	345
per patient, mean (SD)	3.8 (4.8)	3.8 (5.0)	3.5 (4.3)
per patient, median [IQR]	2 [1–4]	2 [1–4]	2 [1–4]
Products received (% of patients)			
RBC	168 (48.7%)	99 (40.4%)	68 (69.4%)
PCC	234 (67.8%)	177 (72.2%)	57 (58.2%)
Platelet concentrate	109 (31.6%)	85 (34.7%)	23 (23.5%)
Albumin	108 (31.3%)	77 (31.4%)	30 (30.6%)
Autologous transfusion	97 (28.1%)	74 (30.2%)	22 (22.4%)
Fibrinogen	66 (19.1%)	54 (22.0%)	11 (11.2%)
Laboratory values before administration			
Hemoglobin, g/dl, mean (SD)	7.69 (0.89)	7.72 (0.89)	7.64 (0.88)
Platelets, ×10³/µl, mean (SD)	159 (72)	159 (72)	166 (78)
Quick’s test, %, mean (SD)	69.2 (12.9)	69.9 (11.6)	66.3 (15.3)
Fibrinogen, mg/dl, mean (SD)	195 (65)	214 (60)	158 (68)
Serum albumin, g/dl, mean (SD) ^a^	2.68 (0.55)	2.61 (0.53)	2.79 (0.58)

Laboratory values were screened automatically for unit consistency and clinical plausibility against physician-approved reference ranges. ^a^ Serum albumin values documented on a mg/dl scale were converted to g/dl (n=3 within the displayed statistics). No documented value fell outside the plausibility ranges

In total, 319 distinct SNOMED CT concepts were recorded in the pilot cohort (88 as primary diagnoses, 187 as secondary diagnoses, 68 as interventions/procedures). The most common primary diagnosis was coronary arteriosclerosis (*n* = 65, 18.8%), which was also more prevalent in males (22.0% vs. 11.2% of females). The most frequent comorbidity was hypertensive disorder (*n* = 47, 13.6%). The most common procedure was aortic valve replacement (*n* = 72, 20.9%). Sex-specific differences emerged: aortic valve replacement and bypass grafting were more common in males (23.7% and 11.4%) than females (14.3% and 6.1%), while mitral valve procedures were more frequent in females (replacement 22.4% vs. 9.8% of males; repair 8.2% vs. 2.9% of males, Table S2).

**Fig. 3:**
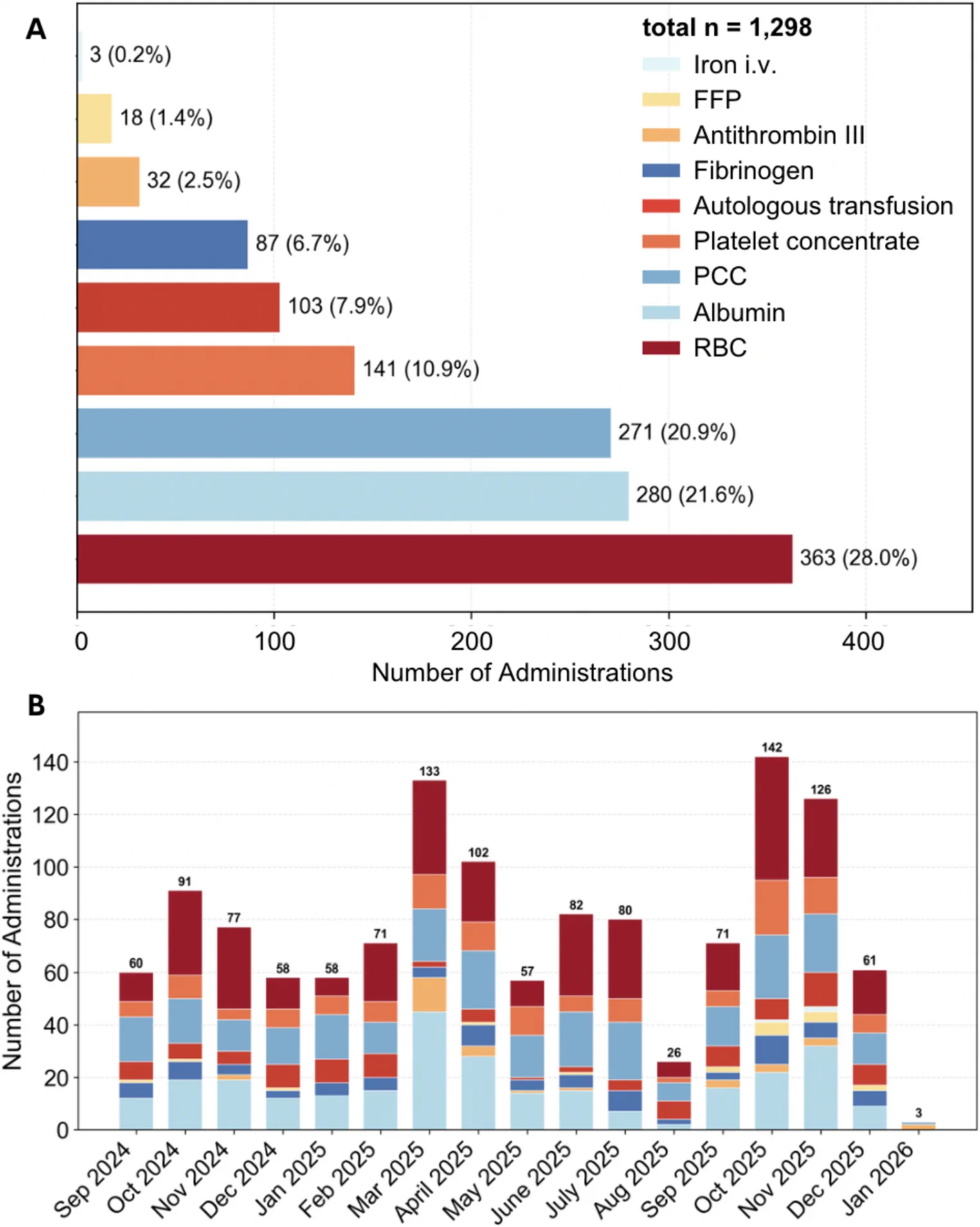
The registry delivers full administration statistics for ten blood therapies; nine had recorded administrations during the pilot period (no oral iron administrations were documented). (**A**) Total administrations by product type (*n* = 1,298) (**B**) Monthly administration volumes by product, colors as in A (*n* = 1,298)

A total of 1,298 blood product administrations were documented. RBC was the most frequently administered product (*n* = 363, 28.0%), followed by albumin (*n* = 280, 21.6%) and PCC (*n* = 271, 20.9%, Fig. 3A). Recorded administration volumes varied across the observation period (Fig. 3B). Total resource consumption during the observation period included 133 L of red blood cell concentrate, 83 L of platelet concentrate, 59 L of albumin, 388,000 IU of PCC, and 239 g of fibrinogen.

**Fig. 4:**
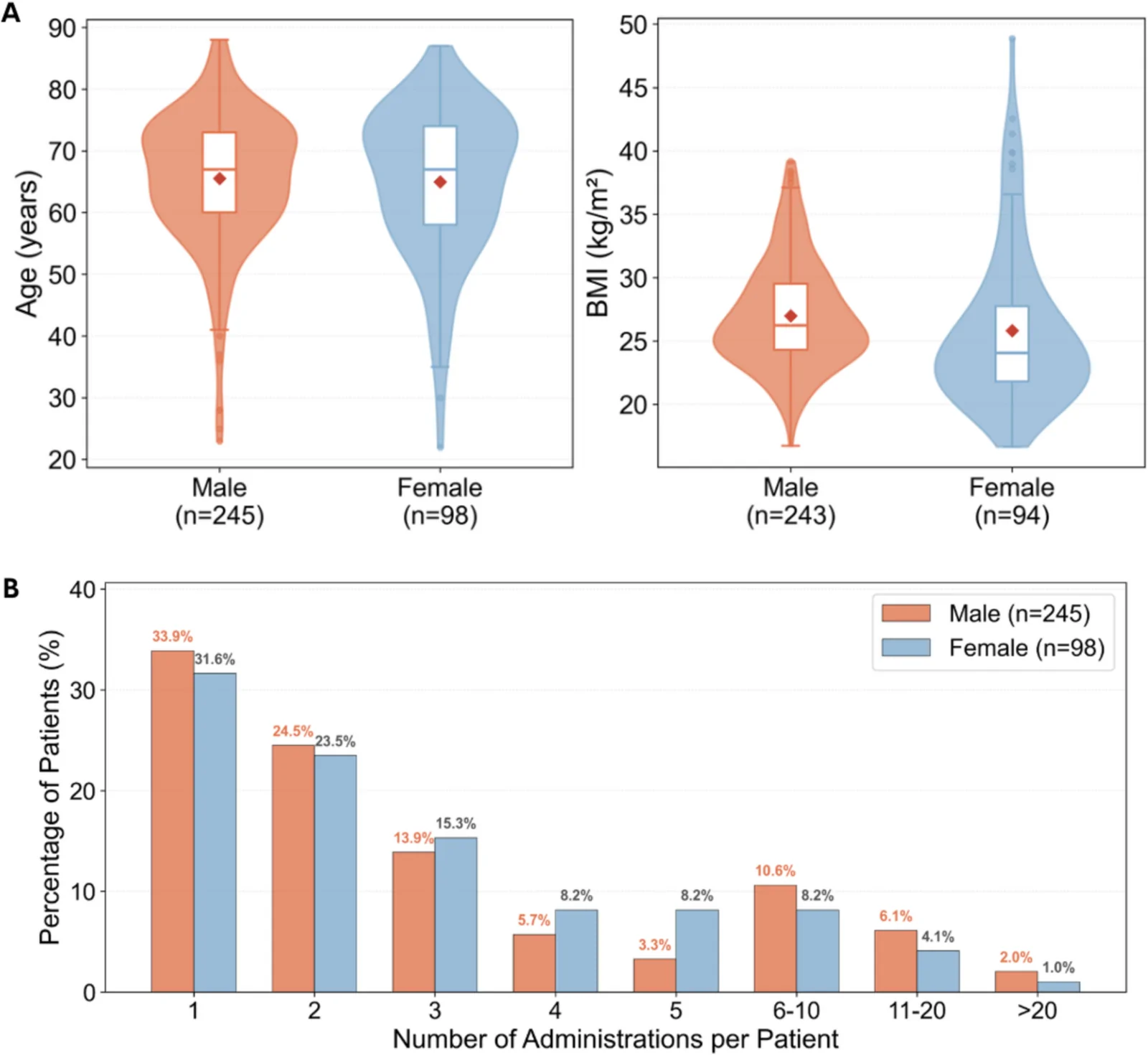
Sex-stratified biometric and therapeutic analysis. (**A**) Age (*n* = 343) and BMI (*n* = 337) distributions. Red diamond indicates mean. (**B**) Blood product administrations per patient (*n* = 343)

The biometric parameters of the patient population such as age (64.3 ± 10.8 years in males vs. 63.7 ± 12.2 years in females) and BMI (27.0 ± 4.1 kg/m² in males vs. 25.8 ± 6.1 kg/m² in females) as well as the distribution of total blood product administrations per patient were comparable (Fig. 4) but sex-specific therapeutic tendencies emerged (Table 1). A higher proportion of female patients received RBC (69.4% vs. 40.4% of males), while male patients more frequently received PCC (72.2% vs. 58.2% of females), platelet concentrate (34.7% vs. 23.5% of females), and fibrinogen (22.0% vs. 11.2% of females). When adjusted for biometrics, dosages were comparable between sexes (Table S3). No records were excluded due to implausible BMI range (see Methods).

**Fig. 5:**
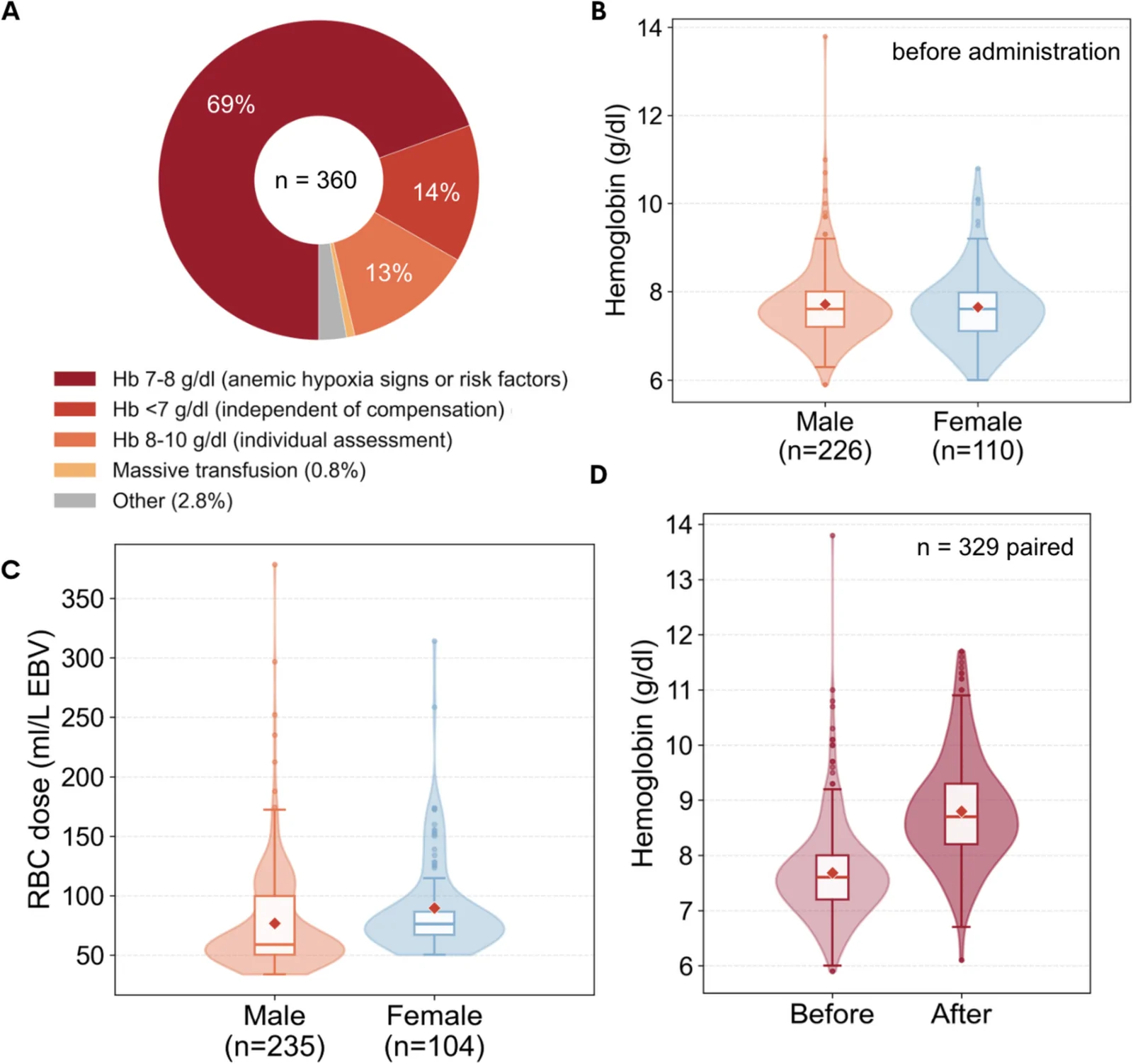
Red blood cell transfusion statistics. (**A**) Indications can be selected in the registry from pre-defined options or entered manually (*n* = 360). Hb, hemoglobin. (**B**) Pre-transfusion hemoglobin by sex shows similar distribution within the study population. (**C**) Dosage distribution for RBC normalized by the EBV according to Nadler’s formula (*n* = 339). Violin densities are estimated over the observed range only; the distributions therefore end abruptly at the observed minima (34.0 ml/L EBV in males, 50.3 ml/L EBV in females), corresponding approximately to a single RBC unit (200–350 ml) relative to the EBV. (**D**) Hemoglobin before and after transfusion shows a rise in hemoglobin by 1.11 ± 0.99 g/dl (*n* = 329 paired). Red diamond indicates mean

To illustrate the scope of the registry, we present detailed statistical data for RBC transfusion (Figure S1). Among the administrations with documented indication (*n* = 360), the most common was hemoglobin 7–8 g/dl with signs of anemic hypoxia or cardiovascular risk factors (*n* = 250, 69.4%, Fig. 5A). Consistently, pre-transfusion hemoglobin was 7.72 ± 0.89 g/dl in males (*n* = 226) and 7.64 ± 0.88 g/dl in females (*n* = 110) (Fig. 5B; Table 1). Mean RBC dose was 76.7 ± 43.6 ml/L EBV in males (*n* = 235) and 89.9 ± 41.6 ml/L EBV in females (*n* = 104) (Fig. 5C, Table S3). In 329 paired measurements, mean hemoglobin increased from 7.69 ± 0.89 g/dl to 8.80 ± 0.99 g/dl after transfusion (Fig. 5D), demonstrating the registry’s capability to capture treatment effects at patient level. RBC was administered to 40–53% of patients undergoing the three most common surgical procedures, which together accounted for 42% of all RBC-transfused patients. Mean pre-transfusion hemoglobin (range 7.2–8.1 g/dl) and dosages were consistent across procedures and sexes (Table S4).

Data completeness varied by product and parameter. Primary and secondary diagnoses were documented in 334 (96.8%) and 224 (64.9%) of the 345 patients who received relevant therapy, respectively. Surgical procedure was documented in 299 patients (86.7%, Table S2). Blood product dosage was recorded for > 84% of the administrations (Table S5). Documentation completeness was lower for laboratory values where routine clinical monitoring is less established, such as serum albumin (44.6%), platelet count (56.0%) or RhD blood group (16.8% of RBC administrations).

The registry includes structured follow-up at 3 months and 1 year after each patient’s first administration. At 3 months, 12.5% of eligible patients responded (*n* = 43/345). Paired baseline–follow-up assessments were available for 20 patients on the CFS and for 21 patients on the MET-based physical-capacity scale. At 1 year, 15.2% (*n* = 47/310) of eligible patients responded. Paired CFS and MET were available for 20 and 14 patients, respectively (Table S6). Complete profiles (baseline − 3 months − 1 year) were available for nine patients on both instruments. 

## Discussion

The Registry Study for Research of Blood Donor and Recipient Long-Term Outcomes is a prospective, web-based framework designed to combine (i) standardized, SNOMED CT-coded documentation of transfusion decisions at the point of care, (ii) risk-adjusted benchmarking, and (iii) patient-reported long-term outcomes linked to these decisions. The results of this 16-month single-center feasibility study in a predominantly cardiac surgery population demonstrate that prospective data collection at the point of care is feasible and yields high-quality data suitable for institutional practice assessment and research. High documentation completeness for key parameters like biometrics, diagnosis and dosage confirms that the eCRF structure supports systematic capture in the clinical setting. Mean pre-transfusion hemoglobin of 7.2–8.1 g/dl across procedures and sexes lies within current restrictive thresholds but does not confirm guideline adherence since no patient-level assessment was performed. A recent review of 61 trials (*n* = 27,639) concluded that future research must recognize the limitations of a single hemoglobin threshold and consider evaluating physiological parameters to guide transfusion decisions [38]. The study framework provides the data to investigate context-dependent transfusion triggers by linking comorbidities, indications, and patient-reported outcomes to transfusion decisions. Sex-specific tendencies emerged in product selection: Women were more likely to receive RBC, whereas men were more likely to receive platelet concentrate, which is consistent with the findings of a retrospective Dutch cardiac surgery cohort study (*n* = 42,388) [39]. In addition, we found that men received more PCC and fibrinogen, an observation that may be influenced by parameters such as the type of surgery, comorbidities, and biometrics and requires in-depth investigation in larger, multi-center datasets. Biometrics-adjusted dosages were comparable between sexes (Table S3).

These observations illustrate the type of practice variation that the designed risk-adjusted benchmarking is intended to detect. It should enable the identification of variations, the distinction between appropriate and potentially inappropriate treatment practices, and the development of hypotheses for targeted quality improvement and research. As the dataset grows and documentation is progressively integrated into clinical workflows, the registry will enable assessment of outcomes in areas where evidence remains inconclusive, such as the clinical significance of AB0 and RhD incompatibility in plasma and platelet transfusions [40]. Follow-up response rates were 12.5% at 3 months and 15.2% at 1 year, potentially creating non-response bias. Even though the respondents resembled the cohort in age and sex (Table S6), other differences cannot be excluded. Standardized implementation benchmarks for surgical PROMs are not established [41], and achieving adequate response rates remains challenging even in optimized outpatient settings [42]. The available paired assessments demonstrate the working data structure for capturing recovery, but they do not yet show that representative longitudinal data can be obtained, and do not support conclusions about recovery itself. Enrollment has continued beyond the pilot period analyzed here: as of 11 September 2026, the registry contains 891 patients with 2,882 documented administrations and is being set up at a second German center [43].

Several limitations should be acknowledged. First, the feasibility study is confined to a single center and a predominantly cardiac surgery cohort; generalizability of documentation patterns, product utilization, and follow-up engagement to other clinical settings and institutions remains to be established. Second, the pilot eCRF did not capture in-hospital mortality; this endpoint has since been added to the eCRF, with data collection ongoing. Third, crossmatch-to-transfusion data, which would quantify resource waste associated with unused blood product reservations, are not currently recorded. Fourth, both the follow-up response rate and the clinical baseline recording of CFS and MET should be improved to obtain more paired assessments of patients’ functional development; this optimization is a priority for the next development iteration.

## Conclusion

This single-center feasibility study demonstrates that a framework for standardized, prospective transfusion data collection can be implemented at the point of care using an open-source registry platform, SNOMED CT-coded data elements, and physical separation of identifying and medical data. Patient follow-up is possible via a fully digital follow-up mechanism based on automated email contact with single-use personalized links to capture PROMs with minimal infrastructural overhead. Improving response rates to counteract potential bias is required for representative long-term outcome analyses. Interoperability with independent systems, the 30-year observation horizon, risk-adjusted benchmarking, and network-wide analyses are design goals that this pilot infrastructure is intended to support. Their validation requires the planned HL7 FHIR implementation and a multicenter network that is currently being established and is open to further institutions.

## Supplementary Information

Below is the link to the electronic supplementary material.

Supplementary Material 1 (DOCX 3.66 MB)

## Data Availability

Individual-level patient data generated in this study cannot be made publicly available, as they constitute sensitive personal health data protected under the EU General Data Protection Regulation (GDPR); participants consented to use within the data governance framework and not to open data sharing. Aggregate data supporting the findings are presented in the article and its supplementary files. The complete analysis pipeline, data dictionary, synthetic validation datasets with documented expected results, and the validation report are publicly available on Zenodo (https://doi.org/10.5281/zenodo.22108007; code: MIT license, documentation and data: CC BY 4.0). A demonstration instance of the registry populated with synthetic data is available on request. Requests for access to de-identified data for legitimate scientific purposes are reviewed by an expert committee under a data-sharing agreement and may be directed to the corresponding author (Jan Andreas Kloka).

## Declarations

### Clinical Trial Number

DRKS00034405 (German Clinical Trials Register, https://drks.de/), registered 10 July 2024; also listed on the WHO International Clinical Trials Registry Platform (https://trialsearch.who.int/).
