# Supplementary material for: A Web-Based Registry Framework Linking Transfusion Practice to Long-Term Patient Outcomes: Design and Single-Center Feasibility Study
Source: J Med Syst. 2026 Oct 21;50(1):153. doi: 10.1007/s10916-026-02474-2 (PMC13683471; doi:10.1007/s10916-026-02474-2)
Supplement: Supplementary file 1 [file 10916_2026_2474_MOESM1_ESM.docx]

**A Web-Based Registry Framework Linking Transfusion Practice to Long-Term Patient Outcomes: Design and Single-Center Feasibility Study**

Elina Nürenberg-Goloub^1^, Benjamin Friedrichson^1^, Michael Nordine^1^, Holger Storf^2^, Kai Zacharowski^1^, Jan Andreas Kloka^1^

^1^ Goethe University, Department of Anaesthesiology, Intensive Care Medicine and Pain Therapy, University Medicine, Frankfurt, Germany

^2^ Goethe University, University Medicine, Institute of Medical Informatics, Frankfurt, Germany

# Supplemental material

**Figure S1: Blood product administrations inclusion scheme for the feasibility study analyses.** The documentation numbers are presented for hemorrhage risk and RBC (Figure 5) analyses. EBV, estimated blood volume; Hb, hemoglobin.

**Table S1: Blood Donor and Recipient Long-term Outcome Study registry feature status.** Designed, implemented and evaluated features of the registry. The evaluation is presented in this pilot report.

| **Capability** | **Status in this pilot** |
| --- | --- |
| Point-of-care clinical documentation (eCRF) | Implemented and evaluated |
| SNOMED CT coding at data entry | Implemented and evaluated |
| Privacy by design (IDAT/MDAT separation) | Implemented and evaluated |
| Automated digital patient follow-up (3-month, annual) | Implemented and evaluated |
| Descriptive institutional statistics | Implemented and evaluated |
| Multilingual platform (16 languages) | Implemented, not evaluated |
| In-hospital mortality endpoint | Implemented after the pilot (added to the basic data form); |
| Risk-adjusted and customizable institutional benchmarking | Designed; requires multicenter network |
| Network-wide analyses (matching, machine learning) | Designed; requires multicenter network |
| Structured data exchange (HL7 FHIR) | Designed (planned OSSE platform feature); not yet implemented |
| Donor enrollment and outcomes | Designed; not part of this pilot |

**Table S2: Five most common diagnoses and procedures stratified by sex.** Diagnoses and procedures with n<3 in any subgroup are not displayed to protect patient privacy. Percentages calculated from patients with documented values.

|  | **Overall (n=345)** | **Male (n=245)** | **Female (n=98)** |
| --- | --- | --- | --- |
| **Documentation coverage** |  |  |  |
| Primary diagnosis | 334 (96.8%) | 237 (96.7%) | 96 (98.0%) |
| Secondary diagnosis | 224 (64.9%) | 158 (64.5%) | 65 (66.3%) |
| >1 secondary diagnosis | 173 (50.1%) | 125 (51.0%) | 47 (48.0%) |
| Procedure | 299 (86.7%) | 210 (85.7%) | 88 (89.8%) |
| **Primary diagnosis** |  |  |  |
| Coronary arteriosclerosis  (SNOMED 53741008) | 65 (18.8%) | 54 (22.0%) | 11 (11.2%) |
| Aortic valve stenosis (SNOMED 60573004) | 59 (17.1%) | 44 (18.0%) | 15 (15.3%) |
| Mitral valve disorder (SNOMED 11851006) | 27 (7.8%) | 16 (6.5%) | 11 (11.2%) |
| Mitral valve stenosis (SNOMED 79619009) | 16 (4.6%) | 9 (3.7%) | 7 (7.1%) |
| Severe mitral regurgitation  (SNOMED 838453008) | 15 (4.3%) | 8 (3.3%) | 6 (6.1%) |
| **Secondary diagnosis** |  |  |  |
| Hypertensive disorder (SNOMED 38341003) | 47 (13.6%) | 37 (15.1%) | 10 (10.2%) |
| Atrial fibrillation (SNOMED 49436004) | 22 (6.4%) | 15 (6.1%) | 6 (6.1%) |
| Coronary arteriosclerosis  (SNOMED 53741008) | 19 (5.5%) | 11 (4.5%) | 8 (8.2%) |
| Heart failure (SNOMED 84114007) | 9 (2.6%) | 6 (2.4%) | 3 (3.1%) |
| **Procedure** |  |  |  |
| Aortic valve replacement  (SNOMED 26212005) | 72 (20.9%) | 58 (23.7%) | 14 (14.3%) |
| Mitral valve replacement  (SNOMED 53059001) | 47 (13.6%) | 24 (9.8%) | 22 (22.4%) |
| Coronary artery bypass graft  (SNOMED 48537004) | 34 (9.9%) | 28 (11.4%) | 6 (6.1%) |
| Mitral valve repair (SNOMED 384641003) | 15 (4.3%) | 7 (2.9%) | 8 (8.2%) |

**Table S3: Normalized blood product dosages by sex.** The eCRF permits documentation in several units per product to accommodate international dosing conventions. RBC and FFP normalized by estimated blood volume (EBV) using Nadler's formula. Normalized dosages include only administrations documented in the product's standard unit at the pilot site, with the biometric data required for normalization. Other products are normalized by body weight (dose per kg).

| **Product** | **Unit** | **Male** | | | **Female** | | |
| --- | --- | --- | --- | --- | --- | --- | --- |
|  |  | **n** | **Mean (SD)** | **Median [IQR]** | **n** | **Mean (SD)** | **Median [IQR]** |
| RBC^a^ | ml/L EBV | 235 | 76.7 (43.6) | 58.8 [50.6-99.7] | 104 | 89.9 (41.6) | 76.3 [67.2-86.6] |
| PCC^a^ | IU/kg | 202 | 19.1 (14.6) | 15.6 [10.1-23.3] | 58 | 16.9 (10.9) | 14.8 [8.4-22.9] |
| Albumin | ml/kg | 182 | 2.7 (1.6) | 2.7 [1.2-3.3] | 92 | 3.1 (1.6) | 3.2 [1.6-4.0] |
| Platelet concentrate^b^ | ml/kg | 103 | 8.2 (3.7) | 8.0 [5.8-10.4] | 25 | 8.6 (3.6) | 9.3 [5.3-9.8] |
| Fibrinogen^a,b^ | mg/kg | 67 | 36.1 (16.8) | 26.7 [23.8-48.3] | 16 | 37.3 (19.8) | 31.8 [23.8-35.7] |
| Antithrombin III | IU/kg | 23 | 12.5 (3.6) | 11.5 [11.1-11.8] | 4 | 20.6 (14.9) | 15.6 [12.2-24.0] |
| FFP | ml/L EBV | 11 | 140.2 (72.1) | 127.4  [100.3-135.4] | 5 | 440.1 (317.3) | 356.2  [172.7-712.3] |

*Excluded were ^a^ doses documented in a unit other than the one shown in this table as non-standard units in Germany (RBC n=1, PCC n=5, fibrinogen n=1) and ^b^ implausibly low doses (RBC n=3, platelet concentrate n=1, fibrinogen n=1).*

**Table S4: RBC transfusion by procedure and sex.** n = patients receiving RBC; RBC/patient = mean administrations per patient; Dose normalized by estimated blood volume (EBV) using Nadler's formula; Hb trigger = pre-transfusion hemoglobin. Values are mean (SD).

| **Procedure** | **Sex** | **n** | **RBC/patient** | **Dose (ml/L EBV)** | **Hb trigger (g/dl)** |
| --- | --- | --- | --- | --- | --- |
| Aortic valve replacement  (SNOMED 26212005) | Male | 19 | 2.7 | 68.7 (32.6) | 7.8 (0.8) |
|  | Female | 10 | 1.6 | 89.5 (62.6) | 8.0 (1.0) |
| Mitral valve replacement  (SNOMED 53059001) | Male | 10 | 3.5 | 84.9 (65.5) | 7.7 (0.8) |
|  | Female | 14 | 1.7 | 91.4 (46.7) | 8.1 (1.1) |
| Bypass graft  (SNOMED 48537004) | Male | 10 | 1.2 | 64.3 (21.8) | 7.6 (0.5) |
|  | Female | 6 | 1.5 | 85.7 (30.8) | 7.2 (0.6) |

**Table S5: Data completeness by blood product.**

| **Product** | **n** | **Dosage** | **Indication** | **Lab before** | **Lab after** |
| --- | --- | --- | --- | --- | --- |
| RBC | 363 | 96.1% | 99.2% | 93.1%^a^ | 91.2%^a^ |
| Albumin | 280 | 100% | 95.4% | 44.6%^b^ | 37.9% ^b^ |
| PCC | 271 | 98.9% | 96.7% | 62.4% ^c^ | 65.3% ^c^ |
| Platelet concentrate | 141 | 92.9% | 92.2% | 56.0% ^d^ | 68.8% ^d^ |
| Fibrinogen | 87 | 100% | 95.4% | 43.7% ^e^ | 55.2% ^e^ |
| Antithrombin III | 32 | 84.4% | 90.6% | 59.4% ^f^ | 34.4% ^f^ |
| FFP | 18 | 88.9% | 94.4% | - | - |
| Iron i.v. | 3 | 100% | 100% | 100% ^g^ | 66.7% ^g^ |

^a^*Hemoglobin;* ^b^ *Serum albumin;* ^c^ *Quick’s test;* ^d^ *Platelets;* ^e^ *Fibrinogen;* ^f^ *Antithrombin activity;* ^g^ *Ferritin*

**Table S6: Follow-up statistics.** CFS, Clinical Frailty Scale.

|  | **3-month follow-up** | **1-year follow-up** |
| --- | --- | --- |
| Eligible patients, n | 345 | 310 |
| Respondents, n (%) | 43 (12.5) | 47 (15.2) |
| Age, years, mean (SD) | 65.1 (10.7) | 62.4 (12.2) |
| Male sex, n (%) | 33 (76.7) | 33 (70.2) |
| Anemia diagnosed (yes), n / n answered | 13 / 38 | 7 / 39 |
| Frailty (CFS), median [IQR] | 2 [2-3] (n=41)^a^ | 2 [2-3] (n=45) |
| Paired frailty (CFS) vs. baseline:  improved / unchanged / worsened | 9 / 9 / 2 (n=20) | 12 / 5 / 3 (n=20) |
| Paired physical capacity vs. baseline:  improved / unchanged / worsened | 15 / 5 / 1 (n=21) | 10 / 4 / 0 (n=14) |

*^a^ n=1 cannot/do not want to answer response is excluded from the calculation*
